# Time use, mobility and expenditure: an innovative survey design for understanding individuals’ trade-off processes

**DOI:** 10.1007/s11116-018-9961-9

**Published:** 2018-12-28

**Authors:** Florian Aschauer, Inka Rösel, Reinhard Hössinger, Heinz Brian Kreis, Regine Gerike

**Affiliations:** 1 Institute for Transport Studies, University of Natural Resources and Life Sciences, Vienna, Vienna, Austria; 2 Institute for Clinical Epidemiology and Applied Biometry, Faculty of Medicine, University Hospital Tübingen, Tübingen, Germany; 3 Provincial Government of Lower Austria, Abteilung Landesstraßenplanung, Sankt Pölten, Austria; 4 Integrated Transport Planning and Traffic Engineering, TU Dresden, Dresden, Germany

**Keywords:** Travel survey, Travel time, Time use, Time use survey, Consumer expenditure

## Abstract

A large amount of information is required to model the complex trade-off processes between travel activities, non-travel activities and budget assignment at the individual level. This paper describes the development of a new survey design, which incorporates components of travel surveys, time use surveys and consumer expenditure surveys in an integrated format, which is expected to deliver a richer data set allowing deeper insights into individuals’ activity and consumption patterns. The survey procedure and the incentives paid, which were necessary to obtain acceptable response rates, are also described. Results from two pilot studies using a trip-based and an activity-based diary format are presented. The paper examines to which extent the diaries have been capable of collecting the required data with high quality and response rates. The innovative “Mobility–Activity–Expenditure-Diary” is introduced and results of the main survey using this design are presented. Travel behaviour and non-travel activities were reported at high quality. Expenditures would require longer observation periods (and preferably not only telephone but also personal support in the survey process) to reduce unsystematic variations and to better capture individuals’ long term equilibrium.

## Introduction

The value of travel time has always been subject to extensive debate in both academia and politics. As savings in travel time are often the major justification for infrastructure investments (BMVBW 2003), numerous studies deal with travel demand models which estimate the willingness to pay for a reduction of travel time, the subjective value of travel time savings (SVTTS). In most cases travelling is not a pleasure in itself, but a necessity to reach a location to engage in more pleasurable and useful activities.

However, not only reductions in travel time, but also improvements of travelling comfort are crucial transport policy measures. In recent years the comfort of public transport vehicles has increased significantly. In-vehicle time has become more entertaining and convenient due to the use of mobile devices which allow us to use our time more productively. These developments let expect that the travel activity itself might be perceived more positively for public transport compared to the car (EC 2015).

These two aspects of travel time, the time loss due to the duration of travel and the valuation of travel as an activity itself are reflected in the two components of the SVTTS: (1) the willingness to substitute travel time for other activities (or value of time as a resource VOR), which is the marginal utility of an additional unit of leisure in the DeSerpa (1973) model and (2) the direct valuation of time assigned to travel (VoTAT), also called the value of time as a commodity (VOC). These two components are expected to vary in different ways, so they should both be known in order to fully understand the effect of transport projects (Jiang and Morikawa 2007). In order to shed light on the individual components, it is necessary to integrate travel decisions into the larger framework of time assignment and consumer’s home production (Munizaga et al. 2008).

A main challenge of a joint time assignment and mode choice model with a microeconomic foundation is the merging of the two different types of models: Travel demand models are typically discrete choice models, e.g. mode choice, route choice or destination choice models, which model the indirect utility of travel decisions (Jara-Díaz 1998) based on random utility theory. Time and budget assignment models are continuous models, which model the direct utility of time use and budget allocation according to a Cobb–Douglas or an additive logarithmic utility function. The combination of discrete and continuous choice modelling approaches is a field of ongoing research (Jara-Diaz and Guevara 2003; Bhat 2008; Habib 2013).

Another challenge is the large amount of information required to model the complex trade-off processes between travel activities and non-travel activities. A prominent example of jointly estimating time assignment and travel decisions is the model developed by Jara-Diaz et al. (2008). Using a Lagrange optimization, they derive four equations (first order conditions) to be modelled in relation to each other:

$$\frac{\partial U}{{\partial X_{j} }} = \lambda P_{j} \quad \frac{\partial U}{{\partial T_{l} }} = \mu \quad \frac{\partial U}{{\partial T_{w} }} + \lambda w = \mu \quad \frac{\partial U}{{\partial T_{t}^{MIN} }} + \kappa_{t} = \mu$$

where *U* denotes the utility; *X*_*j*_ and *P*_*j*_ the amount and price of consumed good *j*; *T*_*l*_, *T*_*w*_ and $T_{t}^{MIN}$ the amount of time assigned to leisure, work, and travel; *w* the wage rate; *λ*, *μ* and *κ*_*t*_ the Lagrange multipliers representing the marginal utility of increasing available money, increasing available time, and reducing the minimum time constraint of travel, respectively. Besides the usual socio-demographic and socio-economic variables, the following information is required for estimating such a model:

- time assigned to travel activities;
- time assigned to non-travel activities, with the activities being subdivided into the categories of unconstrained activities (leisure) and constrained activities (such as personal care);
- budget assigned to goods consumption being subdivided into constrained and unconstrained goods.

Given that the objective is to model detailed trade-off processes between travel decisions, time use and budget assignment, it seems to be important to gain all necessary information from the same individual simultaneously. Moreover, data is required to be collected for an observation period of sufficient length to consider the allocation pattern as a representation of the individual’s long term equilibrium. To the best of our knowledge, no dataset exists which meets all these requirements and there is no survey procedure available to collect these data at sufficient quality and quantity in a diary format. Some travel surveys cover long periods (e.g. Chalasani and Axhausen 2004), but information about non-travel-activities can only be roughly inferred from ‘trip purposes’ and no information about budget assignment is included. A possibility to retrieve all required information is the matching of data from independent time use and expenditure surveys (Jara-Diaz and Rosales-Salas 2015; Konduri et al. 2011), but this procedure yields only probabilistic rather than direct relationships between time and budget assignment. Castro et al. (2012) mentioned that the merits and appropriateness of such a synthetic data generation are debatable and further efforts on obtaining combined data on time-use and expenditure are desirable. In 2009, a new module on time use and consumption has been added to the Longitudinal Internet Studies for the Social Sciences (LISS) panel, administered by the CentERdata (Cherchye et al. 2012). Retrospective information on time use during the past 7 days preceding the interview and household consumption expenditure within the previous month (within the last year for large durables) is collected. Yet this data collection method does not yield information on travel modes of on the level of individual activity episodes and can lead to systematically biased mean values of time use (Browning and Gørtz 2006; Juster and Stafford 1991). Dharmowijoyo et al. (2015) deployed a panel time use and activity diary throughout a 3-week period to capture day-to-day variability and repetition patterns. However, the activity diary was sampled at 15-min intervals, hence short duration activities and trips are not recorded and information on goods consumption is not included.

The goal of this study is to develop a survey design that meets all requirements formulated above and at the same time ensures acceptable response burden and high data quality. Such a survey procedure should have an observation period of at least 1 week, since this period captures the rhythms of most activity types sufficiently and is a suitable compromise between response burden and data requirements (Jara-Diaz and Rosales-Salas 2015; Minnen et al. 2015; Senbil and Kitamura 2009; Zerubavel 1985). A combined data collection approach is meant to take advantage of three currently separate survey techniques:

- *travel surveys* including information about the characteristics and determinants of travel activities such as trip purpose, start and end time, duration, cost, transport modes, location of origin and destination;
- *time use surveys* giving complete information about travel and non-travel activities throughout the day including the types of main and parallel activities, location, start and end time of each activity episode;
- *consumer expenditure surveys* dealing with goods consumption and budget assignment in the short and long run.

We merge these three survey traditions into the innovative “Mobility–Activity–Expenditure-Diary” (MAED). This paper reports about the lessons learned from two pilot studies and the results of the following main survey, which uses the final MAED-design. With this research we hope to contribute to the advancement of methods for collecting data on travel behaviour in the context of individual’s overall activity and consumption patterns.

The remainder of this paper is organised as follows: The state of practice for travel surveys, time use surveys and consumer expenditure surveys is presented in “Current state of survey practice” section. Findings from pilot studies which marked the path towards the final questionnaire design are discussed in “The Mobility–Activity–Expenditure-Diary (MAED) design” section. We first develop the general concept of the MAED based on the state of art and the goals of this study in “Approach to integrating the three survey traditions” section. We then explain the questionnaire designs for the pilot studies in “Findings from pilot studies” section and the final MAED design in “The final MAED design” section. “Survey procedure, response rates and incentives” section describes the survey procedure and the tested incentive schemes. Quantitative results of time use and expenditure patterns are presented and compared with data from national Austrian surveys in “Quantitative results of main survey” section. Conclusions and an outlook on further research are provided in the final section.

## Current state of survey practice

### Travel surveys: the trip-based approach

Methods for surveying travel behaviour have been continuously improved since the 1970s when the first national and municipal travel surveys were implemented. No standards for travel survey methods have yet been established, they vary from country to country (Armoogum et al. 2014). Travel surveys are with very few exceptions cross-sectional surveys with the household being the usual sampling unit and the survey duration being one diary day. The German Mobility Panel (GMP) (Chlond et al. 2015) and the research project Mobidrive are examples of multi-day surveys. Mobidrive succeeded to observe longitudinal travel patterns for a 6-week period using written diaries and intensive respondent support throughout the survey period (Chalasani and Axhausen 2004). The British National Travel Survey covers a period of a week (Taylor et al. 2013).

Most current travel surveys offer different channels for survey participation. Self-administered mail-back questionnaires and telephone interviews dominate. Online questionnaires are often provided but only used by small proportions of the participants. Personal interviews are carried out in some countries (see e.g. Centre for Studies on Networks, Transport, Town Planning and Public Building 2009).

Technology-based surveys are increasingly applied in research projects with mainly non-representative convenience samples (see e.g. Kopp et al. 2015). Armoogum et al. (2014, see also Cottrill et al. 2013; Shen and Stopher 2014) give an overview of pilot studies for the integration of GPS-loggers and smartphones into representative national travel surveys. Technology-based surveys make use of the increased availability of location-enabled mobile devices and aim at an improved accuracy of reported trips in terms of numbers, durations and routes in combination with a reduction of response burden. The lacking representativeness of technology-based travel surveys is a major limitation of this promising survey method so far.

**Fig. 1:**
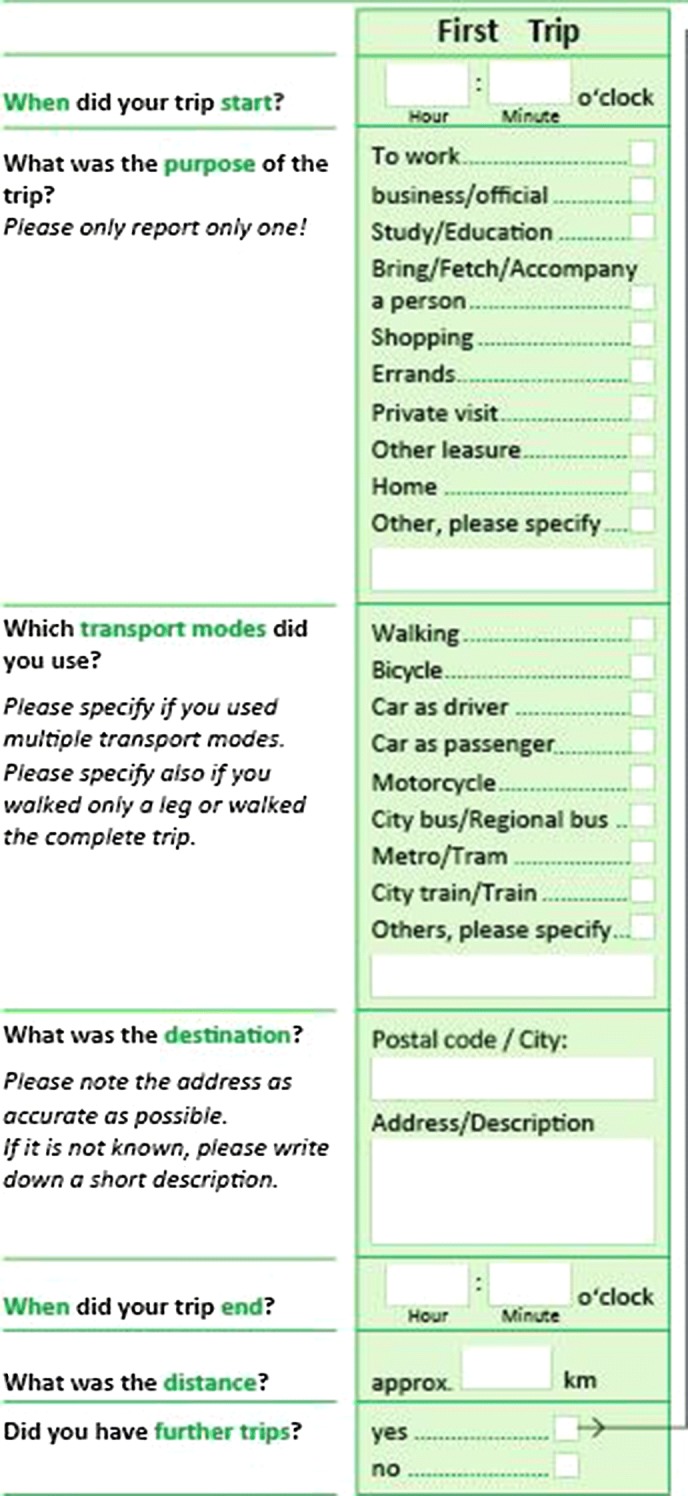
Excerpt of travel diary (*source*: adapted from the Austrian national travel survey 2013/14)

The survey procedure and the written questionnaire design of many travel surveys are based on the New KONTIV-Design (NKD) developed by Socialdata (2009). Households are contacted by mail and motivated via telephone calls. Incoming questionnaires are checked for completeness and additional phone calls are made for validation if necessary. Various reminders and a strict scheduling of all processes are important to achieve high response rates. The NKD-travel diaries list each single trip in one column (see Fig. 1) with usually three to four trips on each page. At least the following data are collected for each trip: start and end time, start and end location, main trip purpose, used transport modes, estimated distance. Further variables such as accompanying persons can be included. Trip purposes are reported within pre-defined categories. The categories vary across different surveys, so far no standards exist. All transport modes used are to be ticked for each trip, but no information about the order, distance or duration of the separate trip stages can be inferred. Travel surveys based on the NKD-design work without incentives, but more burdensome surveys (e.g. with longer reporting periods) use small incentives. Participants in the GMP for instance are offered a lottery ticket. Response rates range from 50 to 80% with the exception of Germany with much lower response rates (Armoogum et al. 2014).

The most recent Austrian national travel survey (NTS) was conducted in 2013/14 based on the KOMOD-guidelines that were specifically developed for this survey (Fellendorf et al. 2011). The KOMOD-survey design heavily relies on the NKD-principles with some modifications, e.g. a 2-day diary instead of a 1-day diary. The data of the Austrian national travel survey will be used for comparison with our results in “Quantitative results of main survey” section.

### Time use surveys: the activity-based approach

Time use surveys (TUS) provide detailed information about the type and location of any activity throughout the entire day. Standards for time use surveys have been continuously developed over almost 20 years resulting in several updates of the guidelines for Harmonised European Time Use Surveys (HETUS) (Eurostat 2004, 2009; UNECE 2013). The HETUS guidelines recommend self-administered mail-back diaries. Current research projects experiment with online questionnaires and mobile devices (e.g. Sonck and Fernee 2013), but national surveys are still mainly based on mail-back solutions. UNECE (2013) lists the use of technologies as one factor that has the potential for recruiting additional person groups and for collecting new types of data.

**Fig. 2:**
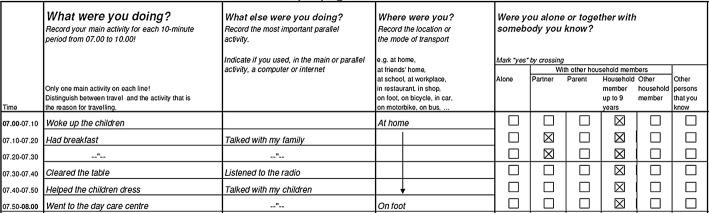
Excerpt of time-use diary (*source*: Eurostat 2009)

Each line in the mail-back written time use diary corresponds to one time-interval of preferably 10 min (see Fig. 2). For each of these intervals respondents are asked to report the main and the secondary activity, the location and additional persons with whom the activity was carried out. Travel is treated similarly to any other non-travel activity type; the question at which location the travel activities take place is to be answered with the transport mode e.g. “on bicycle”, “by car”.

Fixed interval diaries are recommended because of the reduced variation in the level of detail of the reported activities compared to open interval diaries and because open interval data are more difficult to code and process. The time interval varies across different surveys, but most of them are based on 10-min intervals (UNECE 2013). The 10-min interval provides only a rough grid for analysing short trips, but gives a comprehensive overall picture of travel activities and non-travel activities (Gerike et al. 2015). The diary starts at 04:00 a.m. and covers 24 h with 3 h per page.

Attempts have been made to apply time-use diaries which cover a period of seven consecutive days (Glorieux and Minnen 2009), yet according to the HETUS guidelines two diary days should be reported, one weekday and one weekend day. Open text fields instead of fixed activity categories should be used in order to obtain the most comprehensive information possible about what the respondents actually did in each time interval. The HETUS guidelines contain standards for activity classification and minimum lists of activity categories (Eurostat 2009; UNECE 2013). Typical categories for locations are “home”, “work place”, “school”, “other person’s home”, “restaurant”, “hotel”, and “holiday home”. These are usually recorded without geocoding. HETUS time use surveys do not use incentives.

The latest Austrian time use survey was conducted in 2008/2009 by the federal governmental statistical agency Statistics Austria. The survey is based on the HETUS guidelines. The main and parallel activities are reported for 1 day using 15-min (30-min from 11:00 p.m. to 05:00 a.m.) intervals (Statistics Austria 2011). Data from this survey are used for comparison with the results from this study in “Quantitative results of main survey” section.

### Comparison of trip-based and activity-based survey approaches

The trip-based and activity-based approaches have different strengths and weaknesses in capturing travel activities and non-travel activities comprehensively and reliably. Travel surveys provide detailed information about trips but only limited insights into non-travel activities. We can infer types of non-travel activities only from the trip purposes. We have no information about in-home activities; this concerns the time before the first and after the last trip and persons who report no trips at all on the diary day. Advantages of travel surveys are their clear focus on the movement of travellers and related information including open interval start and end times, locations of origin and destination, transport modes, trip distances, the spatial context, the weather on the diary day, and the availability of mobility tools (such as public transport season ticket, private car, car sharing membership) both in general and on the actual diary day.

Time use surveys are rarely used in transport research because the 10-min interval impedes accurate data collection for short trips and because of missing information about locations, the spatial context and car availability. The activity orientation is, however, superior to the trip-based approach in the sense of placing the travel activities in a context that matches the individual’s way of thinking. Travel itself is in most cases a means to an end; it is the individual’s daily activity schedule which creates the demand for travel. As a result, it is to be expected that respondents will report their activities including travel more accurately and completely compared to trip-based approaches. There is no reason to underreport travel by claiming not to have left home or by omitting individual trips (Gerike et al. 2013, 2015). The activity-based approach additionally allows collecting data on in-home activities and for immobile respondents.

HETUS time use surveys treat travel on the same level as any other activity with the disadvantage of losing detailed trip information compared to travel surveys. In addition, short trips of less than 10 min are not reported at all; subsequent activities at different locations are often reported without a trip in-between. Gerike et al. (2015) found an average of 0.9 location changes without a trip in-between per diary day in the German time use survey of 2002. Respondents in time use surveys receive no instructions about whether to report each transport mode for every single trip stage. In the German time use survey of 2002 respondents tended to report only the main mode and intuitively omitted the short stages, e.g. going by foot. Activity sequences of trips without non-travel activities in-between turned out to be sequences of trips and non-travel activities, in which both of them were merged into one activity episode (Gerike et al. 2015).

### Consumer expenditure surveys

Consumer expenditure surveys provide information about the consumption expenditure of private households to monitor general household living standards, well-being and consumption patterns (To and McBride 2013). These surveys are used to examine the economic and distributional impacts of policies and to revise the weighting of the basket goods in the Consumer Price Index.

No standards exist for consumer expenditure surveys. Their design therefore differs from country to country. Variations refer to the frequency of deployment and to the method of data collection. With few exceptions data collection comprises at least two instruments:

- *Expenditure diaries* Respondents report all their actual expenditures for goods and services in diaries, usually over a period of 14 days. Few diaries exist with diary periods of 7 days, 1 or 2 months. Diaries are filled out either for individual persons or for the entire household. Self-administered paper diaries or online diaries are used.
- *Retrospective interviews, questionnaires* In most countries respondents also report long-term and regular expenditures retrospectively for the last 1, 3, or 12 months. These expenses serve to correct the diary data for costs which do not occur in the diary period, and they ensure that also seasonal and one-time big-ticket items are included. This is indispensable for the calculation of the total consumer expenditures.

Monetary as well as non-monetary incentives are offered; some surveys encourage the use of online diaries through higher incentives than for the paper-based diary (To and McBride 2013).

The Austrian consumer expenditure survey is conducted as a household survey by Statistics Austria every 5 years. The most recent survey was carried out in 2014/15.^1^ For 2 weeks all members of each participating household documented their personal expenses on goods and services either in a paper or online diary. Expenses had to be classified into three parts of the expenditure diary:

- *Part 1: Private garden or farming products for personal requirements.* All home-made agricultural products harvested and consumed within the 14-day period had to be recorded in the diary.
- *Part 2: All expenses made on food and drinks including pet food and visiting a restaurant or cafe*. A pre-defined categorization of food groups was used, so that all costs incurred could be stated by choosing the appropriate product and adding its amount and price. Product groups had a high level of detail (e.g. “wholemeal bread” and not just “bread”).
- *Part 3: All other expenses.* For all other kinds of expenses two pages per day were provided offering one page with pre-defined categories of products (e.g. personal care, clothes, fuel) and an additional page with open text fields where respondents had to specify the purchased items.

At the end of the diary a page was provided to note expenses which tend to be forgotten by respondents (e.g. costs automatically debited from a bank account such as newspaper subscription, mobile phone bill).

In order to categorize the expenses made by the household members the *Classification of Individual Consumption Expenditures by Purpose* (COICOP) was used. This is a recommended classification scheme in Europe to group types of consumer expenditures (Statistics Austria 2011).

The Austrian version of the COICOP is shown in Table 1. It consists of 12 main divisions of expenditures, which are further broken down into six hierarchical levels of increasingly refined sub-aggregates. In addition to the 2-week diary, face-to-face interviews were conducted with the household members, a first one prior to the diary period and a second one afterwards. These interviews covered expenditures on major purchases (e.g. vehicles, vacation trips), running costs which are paid on a regular basis (e.g. rent, insurance) and sporadic costs (e.g. the annual pass for public transport) retrospectively for the last year. Questions which could not be answered in the first interview were clarified in the second along with inconsistencies such as double-reporting of expenses.

**Table 1:** Austrian version of COICOP main divisions (Statistics Austria 2011)

COICOP main divisions	
01. Food and non-alcoholic beverages	07. Transport
02. Alcoholic beverages, tobacco and narcotics	08. Communication
03. Clothing and footwear	09. Recreation and culture
04. Housing, water, electricity, gas and other fuels	10. Education
05. Furnishings, household equipment and routine household maintenance	11. Restaurants and hotels
06. Health	12. Miscellaneous goods and services
	[13. Not for private consumption]^a^

^a^Not included in total consumption

Survey participants were reimbursed with a 50 € voucher for completing the survey programme.

## The Mobility–Activity–Expenditure-Diary (MAED) design

### Approach to integrating the three survey traditions

The three survey traditions described above were combined in order to achieve the goal of collecting data about travel activities, non-travel activities and consumer expenditures from the same individuals for a 1-week period. One week seems to be a good compromise between response burden and accurate representation of the individuals’ long-term equilibrium, because intra-personal variation and routines that follow multi-day cycles can be observed for most activity types and expenditures (Jara-Diaz and Rosales-Salas 2015; Minnen et al. 2015; Senbil and Kitamura 2009; Zerubavel 1985). The challenge was to merge the three survey concepts in a way that keeps the response burden at an acceptable level and at the same time delivers all required information in high quality. For meeting this challenge, we removed everything that is not needed for the described model, merged the remaining contents to a clearly arranged questionnaire, and developed a survey procedure that ensures a high response rate for this questionnaire.

Our reference point to define the data requirements was the model developed by Jara-Diaz et al. (2008). In terms of activities this model requires a distinction between work, freely assigned activities (leisure), travel, and constrained activities for which a certain minimum duration is indispensable. In terms of expenses the model requires to differentiate between freely assigned and constrained goods. The detailed classification schemes of activities (HETUS) and expenditures (CIOCOP) are not required; they can be considerably simplified without limiting the options of modelling. The activity classification chosen for the integrated survey corresponds well with the transport literature that often aggregates trip purposes to subsistence (work, education), non-discretionary or maintenance (shopping, errands, accompanying, care, voluntary, personal care, other) and discretionary (leisure) trips (Gerike et al. 2015).

According to the above discussion both the trip-based and the activity-based approach have their strengths and weaknesses in capturing travel activities and non-travel activities. We thus considered both approaches in our pilot studies. Each survey instrument was modified in order to best serve the purpose of this study. The basic modifications and features are listed in Table 2.

**Table 2:** Features and modifications of the three survey traditions in our approaches

*Activity*-*based approach*
*Open time*-*intervals, pre*-*defined activity classification* All tested designs used pre-defined activity categories and open time intervals—contrary to the HETUS guidelines, which recommend open activity description and pre-defined time intervals. The main ideas behind the open time intervals were (1) to ensure that all trips are reported with correct start and end times, also short trips of less than 10 min; and (2) to reduce the response burden, because our scheme avoids multiple recording of long activity sequences (sleep, work etc.). The idea behind the pre-defined activity categories was to indicate the requested level of detail for the reported activities to the respondents. This should help to reduce unwanted variation in the level of detail, which is the main argument against open time-intervals in the HETUS guidelines
*Accurate separation of travel activities and non*-*travel activities* Literature shows that travel activities and non-travel activities tend to be mingled in activity-based questionnaires (Gerike et al. 2015). We thus tested different designs for motivating respondents to report travel-activities reliably and separately from non-travel activities
*Addresses of visited locations* We provided sufficient space to report the complete address for the start and end location of each trip in all versions of the questionnaires. Complete addresses are essential to georeference the locations visited which is a required prerequisite to obtain information on non-selected alternatives in the mode choice models or to add spatial attributes such as the distance to the next public transport stop. The importance of complete and correct locations was additionally emphasised in the instructions
*Trip*-*based approach*
*NKD*-*design* Our main idea was to stick as close as possible to the NKD design because it has proven to be successful in reporting travel-related information, but to expand the ‘trip purpose’ section in order to retrieve more detailed information about non-travel activities. We tested different approaches in the pilot studies to include non-travel activities between the trips, before the first and after the last trip, but also for diary days without any trip
*Travel costs* Questions about travel costs were included directly in the trip section of the diary from the second pilot study on (public transport ticket, parking ticket etc.)
*Consumer expenditure*
*Reduced level of detail in expenditure categories* Consumer expenditure diaries are very detailed with fine subdivisions of product groups. The model of Jara-Diaz et al. (2008) requires first and foremost a distinction between constrained and freely chosen goods; further distinctions may improve the model, but the number of cost categories that the model can deal with is strictly limited. Our classification of reported expenses was based on the main COICOP divisions with 12 categories shown in Table 1. In pilot study 2, pre-defined expenditure categories were tested against an open description of expenses with post hoc classification by the survey team
*Travel costs as an exception* According to our specific interest in travel, our final scheme includes more detailed questions on travel costs than usual travel diaries and expenditure diaries. Consumer expenditure surveys do not have special interest in travel; the COICOP division “Transport” is treated like any other product group

The next section describes the tested questionnaire designs. “The final MAED design” section explains the final MAED design that was used in the main survey.

### Findings from pilot studies

Several versions of questionnaires were tested in two pilot studies and a pre-test^2^ before the MAED design was finalised and applied in the main survey, which ran from April 2015 to December 2015. In the two pilot studies variants of a travel diary (TD) being enhanced versions of the trip-based NKD and variants of an activity diary (AD) closely related to the activity-based HETUS-design were tested (see Table 3 for sample sizes and response rates). Consumer expenditures were included from the second pilot study on.

**Table 3:** Sample and response rates of the pilot studies on household level

	Pilot study 1			Pilot study 2		
Households in %	Overall (n = 300)	TD (n = 100)	AD (n = 200)	Overall (n = 145)	TD (n = 49)	AD (n = 96)
*Recruitment phase*						
Gross sample size	100	100	100	100	100	100
Not available^a^	35	35	35	29	30	29
Participation rejected	27	33	24	42	43	42
Participation agreed: households received questionnaires	38 (n = 114)	32 (n = 32)	41 (n = 82)	28 (n = 41)	27 (n = 13)	29 (n = 28)
*Questionnaire phase*						
Households returned questionnaires^b^	73	81	70	68	62	71
Net response of gross sample	28	26	29	19	16	21

^a^No phone number, not in target area, failed to contact, no communication possible

^b^Percentages based on households which received questionnaires

The main challenge of the *activity diary* (AD) was to make respondents report trips and activities in separate lines (time segments) of the diary. Apart from the basic modifications described in Table 2 the AD of pilot study 1 was very similar to standard HETUS time-use diaries. It comprised an open textfield for all activities other than those offered in categories and another textfield to record either the location of the non-travel activity or the modes of transport in case of a trip.

**Fig. 3:**
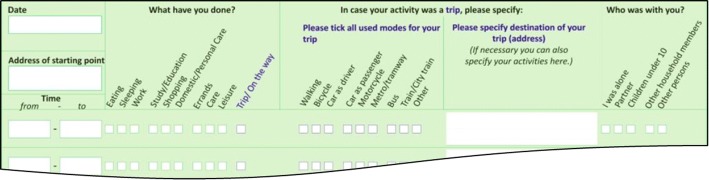
Pilot study 2, activity diary (AD) based on HETUS expanded by travel mode and address

Pilot study 2 had two modifications: (1) The daily 04:00 a.m.–04:00 p.m. scheme was given up for starting the day with getting up. This avoids to artificially split up sleep time into two separate lines, which was overruled by several respondents (see Table 4) and also criticized to cause extra work. (2) It provided a clearer distinction between travel and non-travel activities. The activity category “Trip/On the way” and the related information boxes were highlighted in blue to emphasize that only if the trip box is ticked information on transport modes and trip destination is required (see Fig. 3). Transport modes were offered in categories and the destination of the trip could be stated within the same line, so that the entire trip could be reported in one line, separately from non-travel activities. An additional help sheet with instructions on how to fill in the diary was enclosed which explicitly said not to mingle trips with non-travel activities and to always tick just one main activity within a line.

**Table 4:** Problems occurring for the diary schemes in pilot study 1 and pilot study 2

	Pilot study 1		Pilot study 2	
	AD (n_Diaries_ = 106; n_Trips_ = 2669)	TD (n_Diaries_ = 50; n_Trips_ = 1329)	AD (n_Diaries_ = 43; n_Trips_ = 991)	TD (n_Diaries_ = 20; n_Trips_ = 487)
Occurrences within diaries in %				
Mixture of trips and activities within one line	35	_	51	_
More than one activity category were chosen	21	_	53	10
04:00 a.m.–04:00 p.m. scheme disregarded	8	_	_	_
Inconsistent durations	_	8	_	_
Problems with activities from 04:00 until first trip	_	4	_	_
Return trips missing, legs instead of trips	_	2	2	_
Occurrences within trips in %				
Missing modes	4.2	1.7	0.2	0
	χ^2^ = 17.04 (*p* < 0.0001)		G = 1.59 (*p* = 0.2062)^b^	
Missing addresses	7.9	5.2 (n = 1664)^a^	12.7	4.9
	χ^2^ = 10.3 (*p* = 0.0013)		χ^2^ = 18.16 (*p* < 0.0001)	

^a^Including missing start addresses prior to the first trip of the survey week

^b^Log Likelihood ratio statistic (G) instead of Chi squared due to small counts

**Fig. 4:**
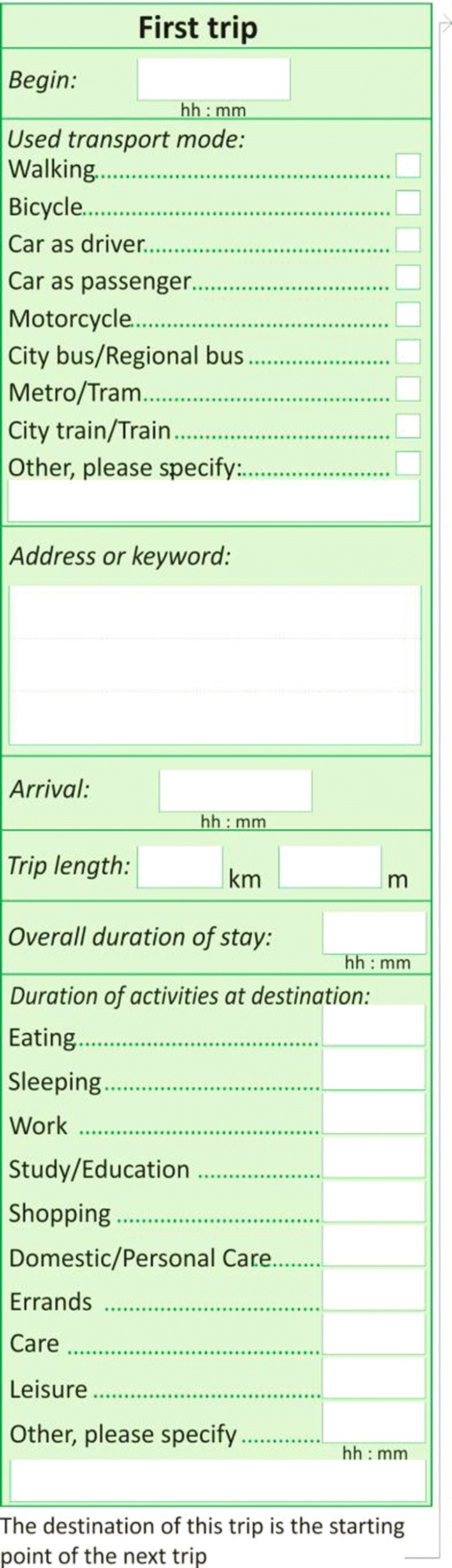
Pilot study 1, travel diary (TD)

The *travel-based diary* (TD) was tested in two versions. Figure 4 shows the version of pilot study 1. The questionnaire design was very close to the NKD design: three trips per page were displayed in columns with the usual travel information such as transport modes, location of the destination, start time, arrival time and trip length. Only the trip purpose section was modified to a list of pre-defined activity categories.

Each category had a text field to report the total duration spent for this kind of activity at the destination of the trip. Asking for the total duration instead of the start and end time of each activity episode was meant to reduce response burden, however at the expense of losing the information about frequency and duration of single activity episodes. Most respondents managed to report the duration of non-travel activities consistently with the start and end times of the trips, but complained about the burden resulting from this calculation. 8% of the respondents reported inconsistent durations for non-travel activities; 4% did not correctly report the durations of the activities between 04:00 a.m. and the first trip, 2% forgot to state return trips or split up trips into legs.

The TD design of pilot study 2 featured a list for chronological sequencing of activities adjacent to the column for the trip information in order to relieve respondents from cumbersome calculations. The activity section appeared very similar to the version used in the AD with pre-defined activity categories and an additional question on accompanying persons. The bottom of the trip column provided questions on selected transport expenses linked to the trip. This design caused only minor inconsistencies with small time gaps between the arrival time of a trip and the start time of the next activity, which could easily be corrected in the process of data entry.

A great proportion of the respondents who filled in an AD mingled travel and non-travel activities within one line. In the first pilot study 35% of the respondents reported travel and non-travel activities systematically in a wrong way: The whole sequence of trip, activity at the destination, and return trip was stated within one line (e.g. trip to the shop, shopping, and trip back home). It was assumed that this occurred because a trip is perceived as something directional resulting in a location. To state the transport mode instead of the trip destination seemed to have been misleading. In such cases it was impossible to code the activities properly, because neither the destinations, nor the transport modes and travel times could be identified. As a consequence, one-third of the questionnaires could not be used for further analyses. The attempt to solve this problem in pilot study 2 and to clarify the scheme was not successful, 51% of the respondents still made the same mistake.

When *comparing the activity diary with the travel diary*, the most serious disadvantage of the AD is that it caused a large number of respondents to tick more than one activity category in one line (21% in pilot study 1 and 53% in pilot study 2) despite the instruction to always choose just one main activity per time segment. In the TD of pilot study 2 only 10% of the respondents ticked more than one activity category, although parallel activities were allowed if the respondent could not decide for just one main activity. The lower part in Table 4 shows the analysis of missing modes and trips in both pilot studies. The TD data exhibited significantly fewer missing addresses than the activity diary in both pilot studies. The same applies for missing modes in pilot study 1. With the improved design of the AD, missing modes were hardly a problem in pilot study 2.

**Fig. 5:**
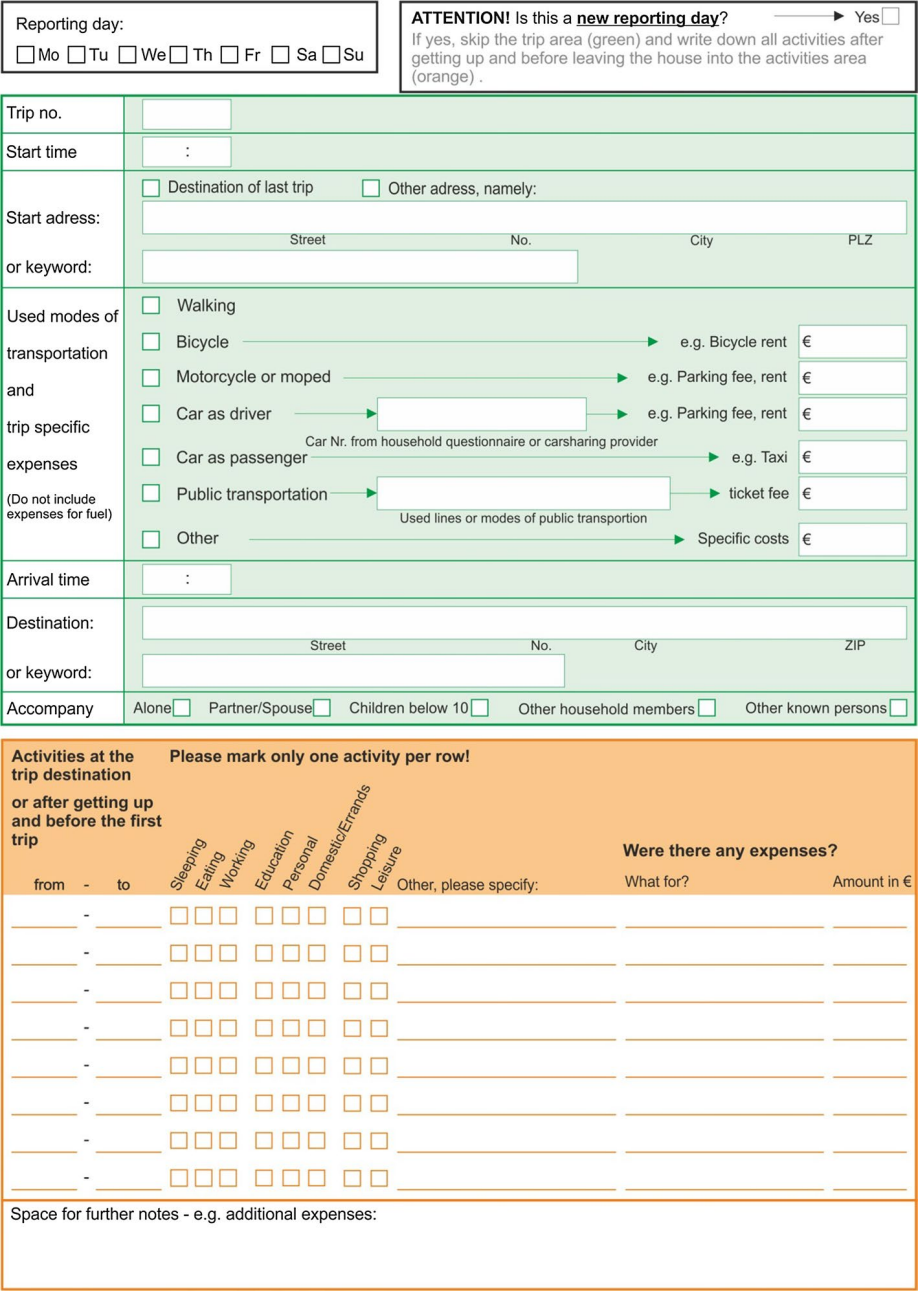
The final MAED design used in the main survey

Overall, the trip-based diaries performed better than the activity-based versions. The HETUS-based questionnaires is intuitive and simple, but trips are not well reported. If additional information on trips is included it becomes complicated and misleading. These findings are confirmed by other studies that also report missing or inconsistent trips for HETUS diaries (Gerike et al. 2015). Structuring the day by trips and asking for non-travel activities between those trips in a second step proved to be more self-explanatory. In addition, respondents who filled in the activity-based diary reported a longer duration for completing the diary (30–40 min compared to 20 min for the travel diary) and stated to be less willing to extend the reporting period in return for a higher incentive than those who filled in a travel diary. As a consequence, we used the trip-based design as a basis for further development of the MAED, which was subject to the pre-test and, after slight alterations, was used in the main survey (see Fig. 5).

The *expenditure diary* was included in pilot study 2 for the first time. The questions about expenses were provided on an extra sheet (separate from travel activities and non-travel activities) at the beginning of the diary (in the AD version) or at the end of each diary day (in the TD version). We tested two versions of expenditure questions: one with an open description of the expenses and one with pre-defined categories according to the COICOP main divisions.

The expenditure descriptions in the open design could be readily interpreted; most stated expenses could subsequently be assigned to a COICOP category. Around 11% of the expenses could not be assigned, but these were no missing values. They included statements such as ‘present’ or ‘pocket money’ for children. The advantage of the open design over the pre-defined categories is that even if it is not known what kind of goods these expenses were spent on (food, clothing, culture, restaurant etc.), the statements are still meaningful in terms of their assignment to committed and freely chosen expenses. The pre-defined categories bear a higher risk of misinterpretation. They may lead the respondents to choosing the wrong category without noticing the mistake. The share of expenses differed considerably for the categories ‘food’ and ‘restaurants and accommodation’ between the design with open categories and the design with pre-defined categories. The open descriptions enabled a clear identification of expenses associated with visits to a restaurant, which were assigned to ‘restaurants and accommodation’; respondents with a pre-defined cost sheet may have categorised such expenses as food. This supports the assumption that pre-defined cost categories can have different meanings for different individuals. So even though the effort to categorize openly reported expenses is high and interpretations depend on the coding person, the open design better suits our requirement to make a distinction between freely chosen and committed expenses.

### The final MAED design

A sample page of the final MAED design is shown in Fig. 5. It is simpler and more self-explanatory than the pilot versions: Each trip is reported on a separate page and each page is divided into two boxes: the upper box contains the travel section based on the conventional NKD; the lower box contains the activity section based on our simplified activity diary design developed during the pilot studies. Each activity being performed at the destination of the trip is reported line by line.

A diary day starts with getting up in the morning and ends with getting up on the next morning. This diurnal division is more intuitive than the 04:00–04:00 scheme of time-use diaries, which in most cases artificially divides the sleeping period into two blocks before and after 4:00 am. A new diary day starts on a new page, but in this case the trip section is skipped and all in-home activities after getting up and before the first trip are reported in the activity section. Thanks to this approach, the MAED features a continuous scheme of uniform pages for the entire week. It is not necessary to provide a pre-defined number of pages for each single day, but only enough pages for the whole week. This reduces the total number of required pages by half. The final MAED includes 51 diary pages for seven consecutive days. The number of pages corresponds to the maximum number of trips reported by participants in the pilot studies including a buffer.

The *trip section* includes the following information for each trip: start and end time, address of start and end location, the transport modes used and accompanying persons. Frequently visited locations (points of interest) can be noted on an extra page at the beginning of the diary with the corresponding address and a keyword (e.g. home, work), so that only the keyword must be stated in the address field of the trip section. The trip section also includes additional information, which is not part of the conventional NKD: (1) in case of car use the reference number of the car which is specified in a household questionnaire; (2) in case of public transport use the line numbers; (3) occasional travel costs (car rental, parking fee, bus ticket etc.) for all modes except walking.

The *activity section* corresponds to the simplified scheme derived from the HETUS diary. Non-travel activities are to be listed chronologically line by line and specified according to pre-defined categories. Activities that do not match a pre-defined category are to be specified in an open text field.

*Questions on expenditures* are included in different parts of the questionnaire. Daily expenses that occur during the observation period are stated directly in the diary pages. The joint statement of activities and expenses is intended to help to remember either of them: stating an activity may bring an expense to mind and vice versa. Travel-related costs are included in the trip section as described above. Expenses related to non-travel activities are stated along with the corresponding activity in the activity section. Following the findings of pilot study 2 that open descriptions are better to interpret and to classify in line with the model requirements than pre-defined cost categories, the diary page provides an open text field to describe the expense (e.g. groceries, cinema ticket, clothes) and another field for the amount. Expenses which cannot be linked to a reported activity (e.g. pocket money for children) can be stated at the bottom of the diary.

Infrequent long-term and regularly recurring payments, which do usually not arise on a weekly basis, are asked in the household questionnaire alongside with socio-demographic variables and available mobility tools (public transport season ticket, private cars, car sharing membership etc.). This scheme is closely related to the consumer expenditure surveys, which acquire additional information about long-term costs retrospectively for 1 year.

The household questionnaire of the MAED covers three segments for expenditures: “rent and housing costs”, “mobility costs” and “other long-term household expenditures”. Expenditures have to be stated for given time intervals (per month, half-year, year) depending on the type of good or service. This is assumed to be more convenient than summing up a monthly paid rent to the annual amount or recalling all actual costs incurred within the last 12 months. Mobility costs include season tickets for public transport, purchases of new vehicles (price and year of latest purchase or leasing rate), and monthly parking fees at home or at work. Pre-defined expenditure categories are applied here that follow the COICOP classification to avoid the risk of respondents forgetting about important categories. Open text fields are provided only for other long-term payments not included in the pre-defined categories.

Due to the fact that the Jara-Diaz model heavily relies on the wage rate (see Jara-Diaz et al. 2008), the population of the MAED survey are households with at least one employed person. Only employed persons were to fill in the diaries, an additional cost sheet was provided for non-employed household members to account for money transfers within a household. In open textfields they could list all their expenses made during the diary week.

## Survey procedure, response rates and incentives

A major challenge of the MAED survey is the high response burden caused by the large amount of information, the complexity of information, and the long observation period. In order to achieve high response rates and data quality it is necessary (1) to motivate the respondents at the beginning and again at crucial stages of the survey, (2) to provide individual support during the reporting period—written instructions are important but not sufficient, and (3) to offer an incentive in return for the high effort. In terms of information channels we used only self-administered mail-back questionnaires with telephone announcement and support. We decided against online questionnaires for several reasons:

- People tend to use the same channel for their answer through which they have been contacted (BRAWISIMO 2015). If they receive a written announcement they prefer a written questionnaire.
- There is some evidence that online questionnaires are filled out with less care and have more missing data, e.g. a high number of missing return trips in travel diaries (Kadan 2015).
- The survey served as a first feasibility test of the MAED design. The implementation and administration of an additional web-based questionnaire would have been too expensive. In prospective surveys it is yet desirable to offer both a written and online channel to increase response rates.

**Fig. 6:**
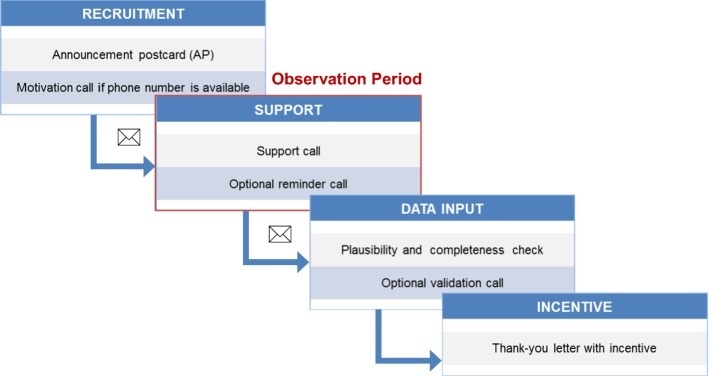
Survey procedure of the MAED survey

The *MAED survey procedure* is based on the NKD (Socialdata 2009). In answer to the higher complexity of information and the higher response burden we integrated additional phone calls. Figure 6 gives an overview of the survey procedure used in the main MAED survey.

The addresses of survey participants were gained from a random selection of Austrian households according to 18 pre-defined strata, which were arranged by region and settlement structure. For around 50% of sampled households a telephone number could be found. The availability of a telephone number makes a difference for the recruitment process, because announcement calls yield much higher participation rates than announcement letters:

- Households with available telephone number (V1) were sent an announcement post card, which notified them that they will be called within the next few days; they were not asked to reply to the announcement.
- Households without available telephone number (V2) were sent a folding card, which informed them of the survey and the incentive in case of participation. They were asked to reply either by returning the folding card or via SMS or e-mail. Responding households were further asked to state the number of employed household members and a telephone number.

Households with telephone number were called for motivation 3 days after the announcement postcard was sent. They were informed about the study and the incentives, and were asked to participate in the survey. Households which agreed to participate were sent a questionnaire package with a pre-defined reporting period. On the first diary day households were called again. This call served two purposes: (1) as second motivation and reminder to start with the diary—if they had not started already; (2) to give the participants some key information how to fill out the diary correctly. Interviewees were trained to explain those parts of the questionnaires that were most important or tended to cause difficulties, e.g., how to code frequently visited locations or how detailed activities should be reported. Respondents could also ask questions. Households that returned the questionnaire in due time received no further call. Otherwise they received a reminder call every week until the questionnaire had been sent back or the household refused to participate.

Households without an available telephone number could only take part if they actively replied to the announcement letter. The answer (folding card, SMS or e-mail) should include the number of required diaries and a telephone number. From that point on the household was treated as described above: sending the questionnaire, phone call on the first diary day etc.

The returned questionnaires were first checked for completeness of documents and then entered into a database. This step included a validation with checks for plausibility and missing values on an automatic and manual basis. In case of missing or implausible answers respondents were called again; open questions were discussed and solved step by step together with the respondent to ensure high data quality.

Table 5 shows the sampling of the main survey. The response rate of telephone households (V1) is at a similar level as in the pilot studies, which results from two balancing effects: On the one hand we had an additional selection criterion ‘non-employed households’, what causes a lower rate; on the other hand we had by definition no ‘unavailable households’ in this group, because households of group V1 were shifted to group V2 (households without telephone number) if the phone number was not valid or if contact attempts failed. A comparison of response rates of both groups shows that households motivated by telephone (V1) responded almost three times more often than those without known telephone number (V2) which had to reply actively to the announcement letter.

**Table 5:** Sample and response rates of the main MAED survey

	Main MAED survey		
Households in %	Overall (n = 4997)	V1 (tel) (n = 1942)	V2 (no tel) (n = 3055)
*Recruitment phase*			
Gross sample size	100	100	100
Not available^a^		39	88
Participation rejected		33	1
Participation agreed: Households received MAEDs	17 (n = 865)	28 (n = 535)	11 (n = 330)
*Questionnaire phase*			
Households returned MAEDs^b^	63	62	64
Net response of gross sample	11	17	7
Usable net response of gross sample after validation of MAEDs	10 (n = 490)	15 (n = 299)	6 (n = 191)

^a^Announcement undeliverable or not returned, no communication possible, non-employed household

^b^Percentages based on households which received MAEDs

The *incentives* were an integral part of the survey procedure. In the main study we offered 40 Euros for each completed diary. The incentive was paid after the diary is returned, checked and validated. This ensured the respondents’ interest to stay in contact with us until the data are finally validated and error corrected. In the pilot studies we tested different schemes:

- The amount varied between 30 and 60 Euros. 60 Euros were too much; the response rates and data quality did not increase accordingly. 30 Euros were sufficient for the simpler diary without expenditures in pilot study 1. The expenditures caused a considerable extra effort so that 40 Euros seemed to be the best compromise.
- We also tested a payment in advance. The motivating effect was indeed stronger than the after-payment of the same amount. Nonetheless we decided on the after-payment to the credit of a higher data quality: respondents were better motivated to answer our validation calls before they received the payment.

## Quantitative results of main survey

An import question is whether our combined and condensed scheme measures the same values as the conventional surveys on time use, travel and consumer expenditure do. The combination of the three survey traditions is expected to affect the level of detail only, but not the distribution of main indicators.

In this section, we match our results with official figures of Statistics Austria as a benchmark. The latter had to be adjusted to fit the prerequisite of a population restricted to households with at least one employed person and the requirements in terms of model specifications described in “Approach to integrating the three survey traditions” section. Tables 6 and 7 present the values of some key socio-demographic characteristics of the MAED survey in comparison with the Austrian national travel, time use and consumer expenditure survey. These surveys are used in “Mobility”, “Time use” and “Consumer expenditures” sections for comparative analyses. In view of the MAED survey’s representativeness for the Austrian population of employed persons/households the Austrian national census (*Registerzählung 2011*) administered by the federal statistical agency of Austria (STAT, Bundesanstalt Statistik Österreich) is used as reference. Table 6 shows that women are slightly overrepresented in the MAED survey sample and the age distribution is left-skewed with younger employed persons being underrepresented, especially those aged 20–29. While the ratio of employed and self-employed persons corresponds well to the population the numbers on the highest educational degree attained indicate that more highly-educated people took part in the MAED survey. Graduates of universities, for example, are represented 2.5 times more often than in the Austrian census. As far as mobility surveys are concerned, this is a well-known phenomenon (Gerike et al. 2015). The overrepresentation of university graduates is also due to the higher average age of participants, so they are more likely to have already completed their education.

**Table 6:** Personal characteristics (employed persons) of the MAED survey compared with national surveys^a^

	MAED survey	Statistics Austria National Census 2013	NTS 2013	Austrian Time Use Survey 2008/09
Households	490	2,006,004	10,490	3060
Employed persons	748	4,019,408	17,013	4546
Gender				
Male	50.0	53.3	53	50.0
Female	50.0	46.7	47	50.0
Age				
15–19	2.3	5.0	0.9	3.5
20–29	6.8	19.5	13.6	17.1
30–39	18.7	22.6	19.1	26.8
40–49	35.7	29.1	31.7	30.1
50–59	31.9	20.0	31.2	19.1
60+	4.6	3.8	3.5	3.4
Employed	88.7	88.8	n.d.	89.1
Self-employed	11.3	11.2	n.d.	10.9
Compulsory education	2.7	17.8	5.9	11.7
Apprenticeship, vocational school	36.0	50.9	48.3	60.2
High school	24.3	15.9	20.2	14.5
College, university	37.0	15.4	25.6	13.6

^a^Characteristics of the Austrian Consumer Expenditure Survey 09/10 were not available

**Table 7:** Household characteristics (households with at least one employed person) of the MAED survey compared with national surveys^a^

	MAED survey	Statistics Austria National Census 2013	NTS 2013	Austrian Time Use Survey 2008/09
*Household size*				
1 person	14.5	30.2	13.3	15.1
2 persons	29.4	23.1	30.0	27.0
3 persons	22.0	19.0	24.0	22.7
4 persons	27.1	18.2	22.6	24.6
> 4 persons	6.9	9.6	10.1	10.5
Urban	24.1	33.5	26.7	26.8
Intermediate	28.2	29.9	27.9	28.2
Thin	47.8	36.7	45.4	45.0
*Target region*				
Eastern Region	33.9	44.1	47.5	30.2
Upper Austria	23.1	16.9	5.6	15.0
Styria	18.2	13.8	21.9	10.4
Salzburg	6.9	6.4	4.7	15.7
Carinthia	5.1	6.2	4.2	9.0
Tyrol, Vorarlberg	12.9	12.7	16.0	19.7

^a^Characteristics of the Austrian Consumer Expenditure Survey 09/10 were not available

The group of single-person households is underrepresented in the MAED (14.5%), employed single-person households add up to over 30% of Austrian households (see Table 7). The group of households with 4 members, in contrast, is overrepresented (MAED: 27.1%, Austrian national census: 18.2%). Regarding the level of urbanisation response rates were higher in rural areas. This explains to some extent the low number of single-person households, because they are found more often in urban areas. In small municipalities only every fourth household is a single-person household, whereas in cities this applies for almost every second household (Statistik Austria und Österreichischer Städtebund 2014).

The average monthly labour net income of fully employed persons cannot be directly compared due to Statistics Austria’s missing objective definition of the term ‘full-time job’. The monthly mean net income of fully employed persons (who classified themselves as such) is € 1836 in the Statistics Austria sample, whereas MAED respondents (who worked at least 37.5 h per week) reported € 2309 on average. This difference in income can mostly be explained by the higher level of education of MAED respondents (spearman correlation between educational level and monthly net income is 0.31, *p* < 0.01).

### Mobility

The most recent Austrian national travel survey was conducted in 2013/14 based on the KOMOD-guidelines (Fellendorf et al. 2011). The KOMOD-survey design relies on the NKD-principles with some modifications, e.g. a 2-day instead of a single-day diary. The NTS offered three options to participate (PAPI, CATI, CAWI). As the survey was conducted over the period of 1 year, there is no seasonal distortion. A weighting procedure was performed on the data in order to represent the average daily mobility of the Austrian population. To ensure comparability with the MAED survey, the NTS data were filtered (1) for employed persons and (2) for survey data from the matching survey periods April to June and September to December.

Table 8 displays a comparison of the most important mobility figures of both surveys. Participants of the MAED survey reported a higher level of tripmaking (share of mobile persons per day) than participants of the NTS survey. Especially on working days the proportion of mobile persons is significantly larger. Also the mean number of trips per day and mobile person is higher (3.80 vs. 3.36). Allowing for the type of day (working day, Saturday, Sunday) the MAED results display typical trip rates (see Armoogum et al. 2014): the highest rate on working days (3.98), a slightly lower rate on Saturdays (3.74) and considerably fewer trips on Sundays (2.85).

**Table 8:** Mobility indicators

	MAED survey	NTS 2013	χ^2^	*p* value
	n = 748 persons n = 18,203 trips	n = 9436 persons n = 57,044 trips		
*Share of mobile persons*				
Working day	0.97	0.91	162.1	*p* < 0.0001
Saturday	0.89	0.82	15.9	*p* < 0.0001
Sunday	0.71	0.69	1.82	*p* = 0.257
*Number of trips per mobile person*				
Working day	3.98	3.39	8.09	*p* < 0.0001
Saturday	3.74	3.40	1.78	*p* = 0.074
Sunday	2.85	3.15	− 1.43	*p* = 0.154
*Distance of trips* (*km*)				
Per trip	12.1	14.9	− 8.06	*p* < 0.0001
Per day	45.9	51.9	− 3.47	*p* = 0.0005
*Duration of trips* (*min*)				
Per trip	23.9	25.6	− 3.98	*p* < 0.0001
Per day	90.8	89.5	0.56	*p* = 0.576
*Mode choice*				
Public transport	10.9	11.9	7.36	*p* = 0.007
Car	69.5	70.1	0.44	*p* = 0.507
Bicycle	5.8	5.5	3.25	*p* = 0.072
Walk	13.8	12.5	4.61	*p* = 0.032

The average trip distance of the MAED survey is 12.1 km compared to 15.1 km in the NTS. The higher number of diurnal trips of the MAED survey doesn’t balance out the total mean daily trip distance per person, which is still 6.0 km longer in the NTS. Similarly, the average trip duration in the MAED survey is lower by 1.5 min, but exceeds the total daily trip duration by 0.6 min because of the higher trip rate. These differences may to some extent result from different methods of data collection: NTS trip distances and durations were estimated by the respondents, while we extracted this information from the Austrian traffic information system (VAO) based on geo-coded departure and arrival locations.

The differences in the shares of mode choices between the two surveys are negligible. The share of walking trips is slightly higher in the MAED survey, while NTS has higher shares of public transport. Because of the specific sample of employed persons only and higher response rates in rural areas, the share of car usage is relatively high in the MAED.

The results suggest that trips have been captured well by the MAED survey. The higher share of mobile persons and the higher trip rate of mobile persons indicate that trips were recorded with higher accuracy than in the Austrian NTS (see Table 8). By having to state all non-travel activities subsequently to the travel activities and all expenses linked to these activities, respondents seem to be less likely to forget about trips during the day or to omit short trips deliberately.

### Time use

**Fig. 7:**
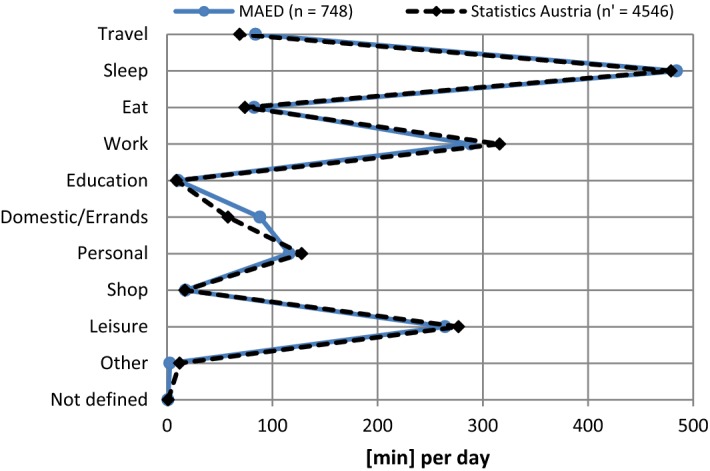
Distribution of time assignment (employed persons) in the main MAED survey compared to the Austrian Time Use Survey 2008/09

Figure 7 shows a comparison of the mean time assignment per day in the MAED with the figures of the Austrian Time Use Survey filtered for the subset of employed persons (see Tables 6 and 7 for sample characteristics).

The results of the MAED fit the time distribution of Statistics Austria very well apart from minor differences which can be explained by coding artefacts. With a differentiation of 426 categories the activity classification of Statistic Austria is much finer and had to be matched with the 10 categories of the MAED. All activities in the MAED which were marked ‘Other’ were coded a posteriori if a further specification of the activity was given or they were classified as ‘Not defined’ if no description was available. In line with home production theory a distinction was made between household production activities such as cooking, cleaning and childcare, which could potentially be outsourced to someone else against payment (e.g. cleaning aid, babysitter) and personal activities which are either bound to the specific person (e.g. being sick at home, visiting a hairdresser) or are usually not transferred to someone else. The former activities were grouped into the category ‘Domestic/Errands’, the latter into ‘Personal’. The surplus of ‘Domestic/Errands’ activities in the MAED may be due to the fact that survey participants were not always able to make a distinction between personal and domestic activities. The slight surplus of ‘work’ in the data of Statistics Austria can be explained by the survey’s distributions of working days which add up to 76% of all reporting days whereas in the MAED survey the share of working days amounts to only 71%.

### Consumer expenditures

Integrating information on consumer expenditures into the MAED posed a major challenge. Whereas time use is restricted to match exactly 168 h every week, expenses vary considerably; buying patterns in a randomly selected week can be quite distinct from the individual’s long-term equilibrium. Moreover, the buying rhythms of goods and services underlay strong variations and cover several orders of magnitude. The questions on expenditures were thus included in different parts of the questionnaire as stated in “The final MAED design” section: the diary (D) focussed on frequently purchased items, the household section (H) on long-term expenditures.

A major issue in this context are exceptional large purchases during the reporting week. Such expenses were allocated to longer time periods according to operating life expectancies. On the other hand, the diary includes some zero spendings for essential consumption categories, for which zero expenses cannot be assumed in the long-term equilibrium, in particular in the categories ‘Food’, ‘Clothing’, ‘Leisure’, ‘Travel’, ‘Services’ and ‘Insurance’. The zero spendings may partly result from the short observation period. We dealt with this problem of under-reporting by imputation, i.e. we replaced zero spendings in essential categories by average expenditures depending on income and household size classes.

Another issue is the overlap in coverage between expenses in the diary and the household questionnaire, because many expenditure categories were addressed in both sources (those labelled ‘D, H’ in Table 9). The overlap requires a procedure of determining the appropriate (more reliable) source or how to combine both sources in a manner that avoids double-counting. We applied two alternative methods:

**Table 9:** Classification of committed and non committed expenditure categories

Category	Classification	Sources
Housing	Committed	H
Food	Committed	D
Accommodation and restaurants	Non-committed	D
Clothing	Non-committed	D, H
Furnishing, household equipment	Committed	D, H
Health	Committed	D, H
Travel	Committed	D, H
Electronics and communication	Non-committed	D, H
Leisure, recreation, culture	Non-committed	D, H
Education	Committed	D, H
Services	Committed	D, H
Financing	Committed	D, H
Insurance	Committed	D, H
Savings	Non-committed	H
Other	Non-committed	D, H

*D* diary, *H* household questionnaire

- Method 1 is based on a source selection method described by Creech and Steinberg (2011) in the Consumer Expenditure Survey Anthology by the U.S. Bureau of Labor Statistics. The Consumer Expenditure Survey (CE) also consists of two instruments, a diary survey for all expenses incurred over a 2-week period and an interview survey that captures expenses for a recall period of 3 months or longer. In order to deal with the significant amount of overlap and to select an appropriate source, the Personal Consumption Expenditure (PCE) estimate produced by the Bureau of Economic Analysis (BEA) is used as reference for a comparison. A Mean Squared Error (MSE) is calculated by adding the variance of the CE data to the squared difference between the mean of the CE data and the PCE estimate. This is performed for both CE sources and the source with smaller MSE is chosen for each expenditure category. We adapted the method for a comparison with the Austrian Consumer Expenditure Survey estimates. The expenses stated in the household questionnaire have consistently smaller variations than those in the diary, so that the decision was always in favour of the household source. However, the smaller variation does not necessarily indicate a higher reliability; it results from the fact that the household section comprises averaged estimates, whereas the diary comprises actual costs with higher variation but lower risk of biased perception.
- In Method 2 we did not select a particular source but calculated the weekly mean values (MV) of diary and household expenses for each overlapping category. This method has two advantages: (1) it reduces the number of zero spendings due to mutual completion; (2) it avoids inconsistencies, if the diary includes expenses during the reporting week, whereas the household section states no spendings for the same category.

The weekly expenses of the category ‘Travel’ were calculated in a different way, because travel costs are required at a trip- and mode-specific level for a mode choice model. Public transport costs accounted for PT reduction cards (H) and ticket costs (D); individual transport costs accounted for vehicle purchases (H), parking space rent (H), road toll stickers (H). A second reason for a different handling of travel expenses was that some of the running costs were not reported: trip costs depending on fuel type and vehicle consumption were estimated and imputed. All travel costs described above were summed up and allocated to a weekly basis. The category ‘Savings’ was calculated by subtracting the total expenses from the total income including labour and fixed income.

Table 9 shows a classification of expenditures into committed and non-committed goods. Expenses on goods associated with physical needs or maintenance activities are traditionally classified as committed (Jara-Díaz et al. 2013). People need to eat (Food), take care of their health and a dwelling place (Housing) with equipment (Furnishing, household equipment). Financing and insurance costs are committed as well as services, which are not related to leisure activities. Expenses on eduction and transportation are also regarded as committed (Jara-Díaz et al. 2013; Mokhtarian and Chen 2004).

Food consumed in a restaurant, accommodation costs on holiday trips, leisure and recreational goods are freely chosen expenses and therefore non-committed. Although ‘Clothing’ is at least partially essential we classified this category as non-committed as expenses added up to fairly high amounts which indicates that basic needs are exceeded. Electronics and communication devices are mainly used for entertainment and thus assigned to non-committed expenses. Savings and all other expenditures which were not further specified are regarded as non-committed.

**Fig. 8:**
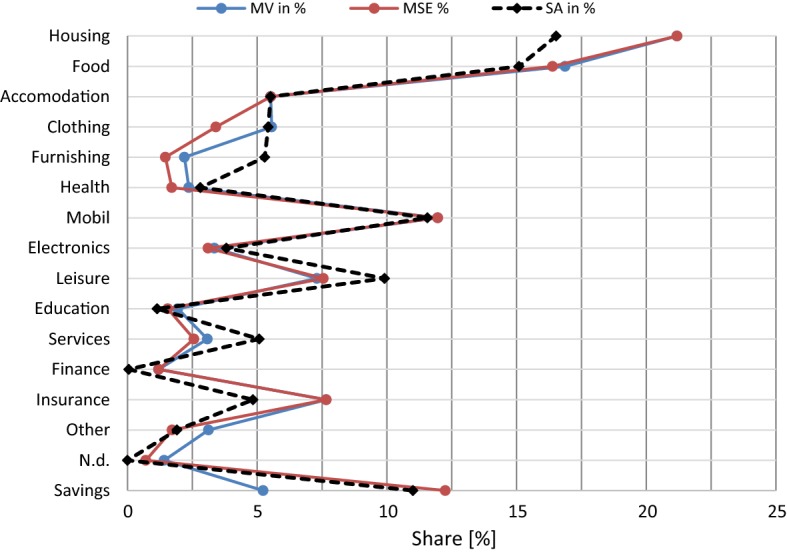
Distribution of expenditure types by calculation methods in comparison with Statistics Austria 2009/2010

Figure 8 shows the expenditure shares of both methods (MSE and MV) in comparison with the Austrian Consumer Expenditure Survey (CES) 2009/10 of Statistics Austria. The dataset of the CES required some processing prior to the comparison: (1) The CES dataset was filtered for employed households; (2) Rental equivalents of owner-occupied housing were removed, as no such values are included in the MAED data; (3) The sub-categories of the COICOP levels were slightly reorganized and recoded to match the MAED categories. This task served mainly to distinguish committed from non-committed expenses. Some inconsistencies remained, thus discrepancies in Fig. 8 can partly result from differences in coding.

Both preparation methods yield similar shares of expenditures by category, and the trends resemble the values of Statistics Austria. One reason for differences may be that the saving rate of private households diminished from 11.3 to 6.9% between 2009 and 2015. However, some expenditure shares display considerable deviations from the values of Statistics Austria, for which no specific explanation is available. We suspect the deviation to be the result of unsystematic fluctuations, which are caused by two factors: (1) the survey period of 1 week was too short to capture the long-term equilibrium with respect to frequently purchased items; and (2) the household questionnaire on long-term expenses was self-administered with telephone support, whereas traditional expenditure surveys include personal interviews where plausibility checks can be performed right away. This seems to be a necessary procedure to obtain more conclusive data.

## Conclusions and outlook for further research

Our motivation for developing a novel survey instrument was to obtain a dataset, which includes all required components to model travel behaviour within the framework of consumers’ home production (Jara-Diaz et al. 2008). To this end we developed a questionnaire and survey design, which enables the collection of data about travel activities, non-travel activities and expenditures from the same individuals over a period of 1 week. The developed survey instrument is based on existing travel diaries, time use diaries, and consumer expenditure diaries. These stand-alone diaries were simplified and re-arranged in order to achieve an integrated MAED with the following features:

- The overall diary structure resembles a conventional travel diary, which structures the day by trips; non-travel activities are nested within the trips.
- Non-travel activities are reported in open time intervals and pre-defined activity categories, although the HETUS guidelines recommend pre-defined intervals and open activity descriptions. This alteration was necessary to keep the response burden at a reasonable level; for the same reason we omitted the parallel activity description, which is a serious downside of this simplification.
- Questions on expenditures are placed within different sections of the diary to achieve an intuitive and self-explanatory scheme: travel expenses in the trip section, expenses related to non-travel activities in the activity section, long-term expenses in the household questionnaire.

It has become evident that the integrated MAED survey performs well and delivers all queried information for travel and non-travel activities at acceptable response rates. Compared to conventional time use and expenditure surveys, it seems that the re-arrangements did not systematically affect the distribution of main activity categories; travel activities are reported more accurately than in conventional travel diaries. Individual telephone motivation and support of respondents as well as an incentive are required in return for the high response burden.

The quality of collected expenditure data is not fully satisfying. We were successful in including all required information in a condensed form into the MAED, however, conventional expenditure surveys put more effort into data collection by means of personal interviews and longer observation periods. This would also be desirable for the MAED to reduce unsystematic variation and to obtain better representations of individuals’ long term equilibrium. A technology-based version of the MAED could be a reasonable way to introduce automated data processing and balance checks in view of income and expenditures.

Despite this moderate weakness there is no doubt that the MAED survey yields a more accurate and consistent dataset for modelling travel behaviour within the framework of consumers’ home production than any existing survey (or the probabilistic merge of different surveys) may yield. We expect an increasing interest in this kind of integrated data, since both travel and non-travel activities have become more flexible and complex, as do the interdependencies between both. Many researchers work on a better understanding the multiple trade-off processes and travel decisions and how they will change in the future. Prominent fields of research are social interactions in multi-person households (Ho and Mulley 2015) and substitutive relationships between in-home and out-of-home activities (Srinivasan and Bhat 2005), which have the potential to heavily affect future travel demand. The research questions evolving in these fields inevitably rely on integrated data that can be obtained through a MAED survey.

## Footnotes

1 Results are so far only available for survey 2009/10.

2 The pre-test will not be further described, as it was very similar to the final MAED survey design.
